# Manipulating the Generation of Photonic Moiré Lattices Using Plasmonic Metasurfaces

**DOI:** 10.3390/nano14020230

**Published:** 2024-01-20

**Authors:** Zhanliang Mu, Yuqin Zhang, Jianshan An, Xuehui Zhang, Haoran Zhou, Hongsheng Song, Changwei He, Guiyuan Liu, Chuanfu Cheng

**Affiliations:** 1 School of Science, Shandong Jianzhu University, Jinan 250101, China; muzhanliang1005@163.com (Z.M.); anjs1999@163.com (J.A.); z17860363721@163.com (X.Z.); zhr20001230@163.com (H.Z.); hshsong@sdjzu.edu.cn (H.S.); changweihe@sdjzu.edu.cn (C.H.); lgy19989@163.com (G.L.); 2 College of Physics and Electronics, Shandong Normal University, Jinan 250014, China; chengchuanfu@sdnu.edu.cn

**Keywords:** moiré lattices, plasmonic metasurfaces, geometric phase

## Abstract

The generation of moiré lattices by superimposing two identical sublattices at a specific twist angle has garnered significant attention owing to its potential applications, ranging from two-dimensional materials to manipulating light propagation. While macroscale moiré lattices have been widely studied, further developments in manipulating moiré lattices at the subwavelength scale would be crucial for miniaturizing and integrating platforms. Here, we propose a plasmonic metasurface design consisting of rotated nanoslits arranged within *N* + *N*′ round apertures for generating focused moiré lattices. By introducing a spin-dependent geometric phase through the rotated nanoslits, an overall lens and spiral phase can be achieved, allowing each individual set of round apertures to generate a periodic lattice in the focal plane. Superimposing two sets of *N* and *N*′ apertures at specific twist angles and varying phase differences allows for the superposition of two sublattices with different periods, leading to the formation of diverse moiré patterns. Our simulations and theoretical results demonstrate the feasibility of our proposed metasurface design. Due to their compactness and tunability, the utilization of metasurfaces in creating nanoscale photonic moiré lattices is anticipated to find extensive applications in integrated and on-chip optical systems.

## 1. Introduction

Moiré lattices can be formed by the superimposition of two (or more) period sublattices that are mutually rotated or twisted with respect to each other. Depending on the angle of rotation, a moiré lattice can exhibit either commensurate (i.e., periodic) or incommensurate (i.e., aperiodic) characteristics, and it is noteworthy that in both cases, the rotational symmetry of the sublattices is preserved by the moiré lattice. Recently, moiré lattices have attracted significant attention in the field of two-dimensional (2D) materials and twisted photonic structures, unveiling a plethora of intriguing phenomena. Magic-angle bilayer graphene has been found to facilitate unconventional superconductivity [1,2,3,4], anomalous quantum Hall effects [5,6] and ferromagnetism [7]. Twisted ɑ-MoO_3_ bilayers exhibit tunable topological phonon transitions from hyperbolic to elliptical dispersions [8]. At a specific rotation angle, these bilayers also exhibit flatband superconductivity [9,10,11] and form atomic photonic crystals [12]. Additionally, stacked transition-metal dichalcogenides display topological excitons among other intriguing properties [13,14]. Compared to 2D electronic materials, the fabrication of 2D photonic moiré lattices through the interference of multiple laser beams offers enhanced design flexibility for moiré-related phenomena. This is attributed to their easy manipulation with various symmetries and twist angles. The broadband light confinement of photonic moiré lattices has been observed [15], along with the enhancement of optical chirality and the generation of laser emissions originating from photonic flatbands [16,17,18]. Additionally, theoretical predictions suggest that these lattices can facilitate magic-angle-induced light localization and hyperbolic plasmons, enabling efficient magic-angle-induced light manipulation [19,20,21,22,23]. So far, several methods have been proposed to produce photonic moiré lattices, such as using a spatial light modulator [24,25], photonic crystal slabs [26,27,28] and a computational hologram [29,30]. These generation methods require large-volume optical paths or complex algorithms. Therefore, new types of photonic devices designed to generate moiré lattices with a small size, high integration levels and multiple functions need to be developed.

Nanoscale moiré lattices offer promising advancements over conventional-sized lattices due to their potential applications in the nanophotonics field. Compared to traditional optical elements and devices, metasurfaces composed of adjustable artificial metallic or dielectric nanostructures possess a remarkable capability to manipulate light fields at the nanoscale and in different degrees of freedom, such as phase, polarization and frequency. They have been successfully utilized for versatile applications including multifunctional metalenses [31,32], holography [33,34] and quantum photon sources [35,36]. The generation of photonic lattices, as one of the most crucial applications of metasurfaces, has been extensively investigated, and various metasurfaces have facilitated the manipulation of photonic lattices. Gao et al. designed a plasmonic metasurface and achieved the generation of a hexagonal vortex lattice with a high integration level [37]. Zhang et al. successfully generated four distinct plasmonic lattices including hexagonal and honeycomb forms by arranging nanostructures on truncated spirals [38]. Tsesses et al., on the other hand, achieved control over lattice topologies by manipulating the dynamic phase through repositioning slit sides or diagonal pairs of slit sides in a hexagon structure, resulting in the skyrmion lattice and its transition from bubble-type to Néel-type [39,40]. However, most previous studies have primarily focused on investigating individual photonic lattices without considering the superposition of two sublattices with specific twist angles to form a moiré lattice.

In this paper, we propose a plasmonic metasurface that incorporates rotated nanoslits distributed within *N* + *N*′ round apertures with different twist angles. The proposed structures can manipulate the superposition of two periodic sublattices at specific angles to generate the moiré lattice at the focal plane. Each individual set of *N* or *N*′ round apertures possesses the capability to achieve a periodic lattice due to multibeam interference. Hence, it is feasible to form a metasurface by interleaving two sets of round apertures in order to realize the superposition of two periodic sublattices. Subsequently, the introduction of a geometric phase through the rotation of nanoslits’ orientation enables the formation of an overall lens and spiral phase and facilitates a controlled phase shift between sublattices. The twist angle between two superposed sublattices is determined by the azimuth position of two sets of round apertures relative to the original point. Consequently, the superposition of sublattices with different twist angles and varying phase differences leads to the generation of moiré lattices exhibiting new period distributions. This method is an advance on previous ones that only allow for one photonic lattice generation and has significance in various applications such as integrated photonic systems, topological photonics and ultrathin quantum metadevices.

## 2. Materials and Methods

**Figure 1:**
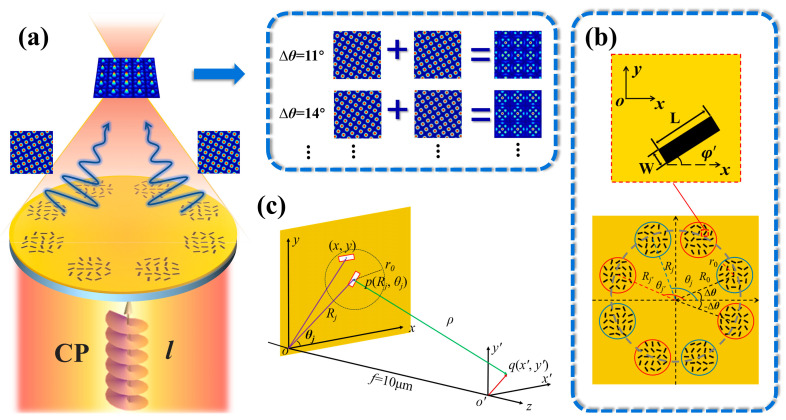
Schematic for generating the focused moiré lattices via a metasurface. (**a**) Basics for the generation of focused moiré lattices. Upon illumination of the incident vortex beam with circular polarization, the output beam is a moiré lattice formed by the superposition of period sublattices. The upper right panel is the illustration of the moiré lattices’ formation in the superposition of two square lattices with Δ*θ* = 14° and 11°. (**b**) Enlarged view of a representative slit and the schematic diagram of the metasurface, and the two sets of round apertures are labeled by red and blue circles. (**c**) Illustrative geometry for the theoretical analysis of the moiré lattice produced by the metasurface.

Figure 1 shows a schematic diagram of the proposed metasurface for manipulating the superimposition of two-period sublattices and generating a moiré lattice in the focal plane. As depicted in Figure 1a, this structure consists of multiple nanoslits that are perforated in a Au film deposited on a glass substrate. Figure 1b presents a top-view schematic of a metasurface sample, where the nanoslits are grouped into *N* + *N*′ round apertures labeled with red and blue circles. The twist angle between the two sets of round apertures is Δ*θ*, with each aperture positioned on a circle of radius *R*_0_. The radial coordinates of the center of the *j*th and *j′*th apertures are *R_j_* and *R_j′_*, respectively, with *j* and *j′* being an integer from 0 to *N* − 1. The azimuthal angles of the *j*th and *j′*th apertures are azimuthally symmetric as *θ_j_* = 2*j*π/*N +* Δ*θ* and *θ_j′_* = 2*j′*π/*N′* − Δ*θ*, respectively. Here, Δ*θ* and −Δ*θ* are the initial angles when *j = j′ = 0*. The position vectors of the central slit are ***R****_j_*(*R_j_*, *θ_j_*) and ***R****_j′_*(*R_j′_*, *θ_j′_*), respectively. These apertures contain multiple nanoslits and are localized into isolated circular regions with *r*_0_, dividing the incident plane wave into multiple separated waves. An enlarged view of a representative nanoslit is presented in the upper part of Figure 1b. The length and width of the rectangular nanoslits are *L* = 200 nm and *W* = 50 nm, respectively, and *φ*′ denotes the angle of the longer side of the slit with respect to the *x*-axis, and thus, the slit orientation angles are defined as *φ* = *φ*′ + π/2. They are designed to form the spiral and lens phase profiles *ϕ_spiral_* (*x*, *y*) and *ϕ_lens_* (*x*, *y*), which vary with the orientation angle and the position within apertures in order to achieve lattice formation and focusing, respectively. A schematic of a metasurface sample is shown in Figure 1a,b, where the arrangement of nanoslits forms eight round apertures, which are grouped into two sets, i.e., 4 + 4, of round apertures. When the incident vortex beam illuminates the sample from the substrate side, a moiré lattice is generated at the focal plane in the output field as a result of superimposing two square lattices at Δ*θ =* 18°. To realize additional moiré lattices, alternative twist angles of Δ*θ* = 14° and 11° were employed, and the resulting intensity patterns are depicted in the upper right panel of Figure 1a.

We now present a theoretical analysis of the wavefield generated by a single rectangular nanoslit located at the center point of the *j*th aperture with position vector ***R*** (*R_j_*, *θ_j_*) in polar coordinates, as depicted in Figure 1c. It is well known that the nanoslit can be regarded as a linear polarizer with the polarization angle *φ* perpendicular to its longer side [41]. The Jones matrix can be written as

(1) $$J\left(\varphi \right)=\left(\begin{array}{cc} \sin^{2}\varphi & \cos\varphi \sin\varphi \\ -\cos\varphi \sin\varphi & \cos^{2}\varphi \end{array}\right),$$

where the slit is illuminated with a circularly polarized (CP) vortex beam $E_{in}^{\sigma }=\left[\begin{array}{l} 1\\ \sigma i \end{array}\right]\exp\left(il\theta \right)/\sqrt{2}$ , with *σ* = ±1 representing left-handed circular polarization and right-handed circular polarization (RCP), respectively, and *l* denoting the topological charge (TP) of the vortex beam. The transmitted wave ***E***_out_ is written as

(2) $$E_{\mathrm{out}}=J\left(\varphi \right)\cdot E_{in}^{\sigma }=E_{in}^{\sigma }/2+\left(E^{-\sigma }/2\right)\exp\left(2i\sigma \varphi \right),$$

where ***E***^−*σ*^ represents the CP component of opposite chirality. It is evident that the transmitted field contains two CP components: one aligns with the incident CP and the other exhibits a reverse CP accompanied by an additional geometric phase 2*σφ*. Thus, the rotated nanoslits provide an efficient way to modify the phase of light in a space-variant manner. For a nanoslit at point (*x*, *y*) on the metasurface, its orientation angle is set as *φ* (*x*, *y*) = *φ_spiral_* (*x*, *y*) + *φ_lens_* (*x*, *y*), and the geometric phase is *ϕ* (*x*, *y*) = 2*σφ* (*x*, *y*) = *ϕ_spiral_* (*x*, *y*) + *ϕ_lens_* (*x*, *y*). Meanwhile, *ϕ_spiral_* (*x*, *y*) and *ϕ_lens_* (*x*, *y*) are given as

(3) $$\phi_{spiral}\;(x,y)=2\sigma \varphi_{spiral}(x,y)=2\sigma (arctan\frac{my}{x}+\varphi_{0})$$

(4) $$\phi_{lens}\;(x,y)=2\sigma \varphi_{lens}(x,y)=2\sigma \pi (\sqrt{x^{2}+y^{2}+f^{2}}-f)/\lambda ,$$

where *m* denotes the rotation order, *φ*_0_ is the initial orientation angle, *f* signifies the focal length, i.e., the vertical distance between the metasurface and observation plane, and *λ* is the light wavelength. Due to the presence of an additional lens phase, only the converted CP component can be focused onto the focal plane*,* thus Equation (1) can be written as [38]

(5) $$E_{\mathrm{out}}=\left(E^{-\sigma }/2\right)\exp\left(2i\sigma \varphi \right)$$

Considering that the radius *r*_0_ of the aperture is significantly smaller than *R_j_*, i.e., *r*_0_ << *R_j_*, the variation in the spiral phase of the nanoslits within the same aperture resulting from positional changes can be considered negligible. Thus, a round aperture can be substituted with a centrally positioned nanoslit to generate an equivalent wavefield. The wavefield ***E_N_*** (*x′*, *y′*) produced by the metasurface on the focal plane is taken into consideration. This field represents the interference among wavelets originating from *N* apertures. Neglecting the size of the isolated apertures, the wavefield in the Fresnel diffraction theory is expressed as

(6) $$E_{N}\;(x^{\prime },y^{\prime })=C\sum_{j=0}^{N-1}\exp\{ik[{\left(x^{\prime }-R_{j}\cos\theta_{j}\right)}^{2}+{\left(y^{\prime }-R_{j}\sin\theta_{j}\right)}^{2}]/2z+il_{1}\theta +2i\sigma \varphi_{j0}\},$$

where $C=\frac{\exp\left(ikz\right)}{\sqrt{2}ikz}\left[\begin{array}{l} 1\\ \sigma i \end{array}\right]$ , *k* = 2π/*λ* and *l*_1_ = (2*σm* + *l*) represent the helical phase that gives the apertures. When the nanoslits are grouped into *N* + *N*′ round apertures, the light field can be written as the superposition of two fields:

(7) $$E_{N+N^{\prime }}\;(x^{\prime },y^{\prime })=C\sum_{j=0}^{N-1}\exp\{ik[{\left(x^{\prime }-R_{j}\cos\theta_{j}\right)}^{2}+{\left(y^{\prime }-R_{j}\sin\theta_{j}\right)}^{2}]/2z+il_{1}\theta +2i\sigma \varphi_{j0}\}\;+C^{\prime }\sum_{j=0}^{N^{\prime }-1}\exp\{ik[{\left(x^{\prime }-R_{j^{\prime }}\cos\theta_{j\prime }\right)}^{2}+{\left(y^{\prime }-R_{j^{\prime }}\sin\theta_{j^{\prime }}\right)}^{2}]/2z+il_{1}^{\prime }\theta +2i\sigma \varphi_{j0\prime }\},$$

where *C*′ = *C*, *θ_j_* = 2*j*π/*N +* Δ*θ* and *θ_j′_* = 2*j′*π/*N′* − Δ*θ* determine the location of each aperture in the metasurface. The above equation indicates that an output field is a superposition of two sublattices, which can be controlled by adjusting phase difference, arbitrary twist angle and spiral order. Obviously, the angle Δ*θ* between the two sets of round apertures allows for flexible modulation of the twist angle of the sublattices. By setting different initial orientation angles *φ_j_*_0_ and *φ_j_*_0′_ of the nanoslits, various phase differences between the two subfields can be also achieved by modulating the spiral order through *σ* and TP *l* of incident light as well as the rotation order *m* of the nanoslit. Therefore, when *N* = *N*′ = 4 or *N* = *N*′ = 6, it is possible to achieve a superposition of two square or hexagonal lattices based on the previous report. By further adjusting the special twist angle and phase difference, diverse moiré lattice patterns are obtained.

## 3. Results

To demonstrate the moiré lattice generated by the designed metasurface, simulations of the transmitted light field were performed using the full finite-difference time-domain (FDTD) method. In our simulations, a CP vortex beam with a wavelength of 632.8 nm was employed and it was incident on the metasurface from the substrate side. To prevent any influence from adjacent periodic cells, an absorbing boundary of a perfectly matched layer was utilized to simulate an aperiodic structure. The radius of the outer circle *R*_0_ was set as 36 μm and the radius of the round aperture *r*_0_ was set as 2.4 μm; the calculation was performed in a transverse area of 50 μm × 50 μm. The Yee cell size used for the computation was 8 nm. The monitors were placed on the air side and focused moiré lattice fields were generated on the plane at *z* = *f* = 10 µm, where the intensities of the converted circular polarization components were observed and analyzed. 

**Figure 2:**
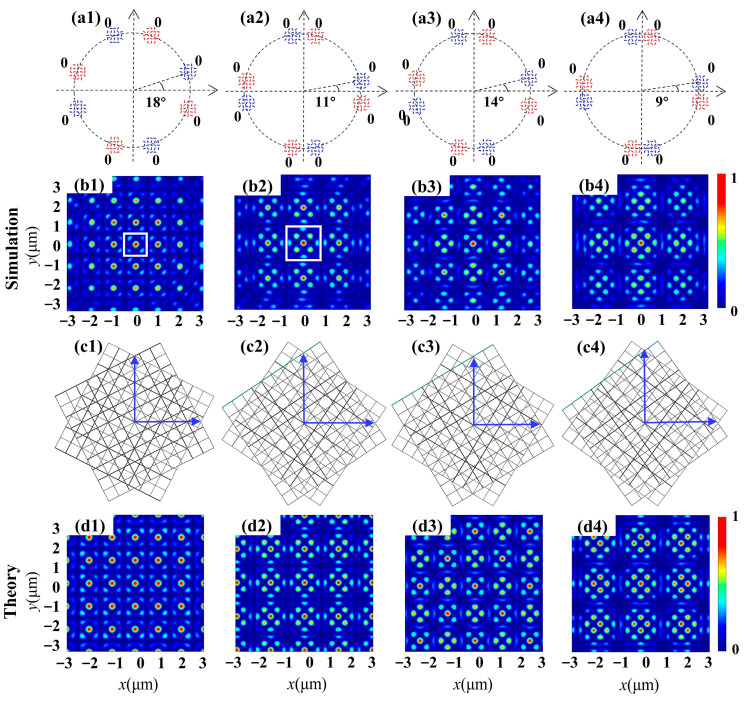
Moiré lattice wavefields generated by the superposition of two twisted square lattices with identical phases Δ*φ* = 0. (**a1**–**a4**) Schematic diagrams of the metasurface sample depict two sets of 4 + 4 round apertures with axes rotated at a mutual angle indicated in each figure, distinguished by blue and red labels. (**b1**–**b4**) Simulated intensity profiles of moiré lattices produced by the interference of two square patterns. (**b1**,**b2**) Periodic lattices and the periodic unit patterns are labeled with different white squares. (**b3**,**b4**) Aperiodic lattices. (**c1**–**c4**) Schematic discrete representation of two rotated square sublattices. (**d1**–**d4**) Theoretical results of intensity distributions.

With the first type of metasurface, the superposition of two square lattices with different twist angles but identical phases is investigated. The intensity patterns of the transmitted fields examined via theoretical calculation and FDTD simulation from the metasurfaces are shown in Figure 2. Here, the designs of four metasurface samples are schematically shown in the first row, where the nanoslits are grouped into two sets of four round apertures, as indicated with the colors red and blue. The slit-rotation order is *m = m*′ = 2, the initial angle of the slit is *φ_j0_* = *φ_j0′_* = 0 and the incident light is an RCP (*σ* = −1) vortex beam with TP *l* = 4. With the illumination of incident CP, the geometric phase 2*σmθ* = −4*θ* induced by nanoslit rotation can counteract the spiral phase *lθ* = 4*θ* carried by the vortex beam, enabling the interference of multiple plane light waves with identical phases. Previous reports have indicated that the interference resulting from the arrangement of four round apertures in a ring can generate a square lattice. Consequently, by employing two sets of four round apertures, it became feasible to superimpose two square lattices to produce a moiré lattice. Further, the twist angles between two sets of four round apertures were adjusted, and different moiré lattices with varying period unit distributions were achieved. The simulated intensity profiles of moiré lattices produced by the designed metasurfaces are presented in the second row. To visualize the formation of superposed fields, the corresponding moiré lattices are displayed in the third row. It can be seen that the results of the FDTD simulation are consistent with those of the theoretical calculations, as given in the fourth row. For the two-dimensional moiré lattices composed of two square lattices rotating with each other, only when the rotation angle Δ*α* satisfies cos (Δ*α*) = *a/c* and sin (Δ*α*) = *b/c*, where the positive integers (*a*, *b*, *c*) constitute a primitive Pythagorean triple *a*^2^ + *b*^2^ = *c*^2^, do the photonic moiré lattices exhibit periodicity. Such angles are hitherto referred to as Pythagorean. For other rotation angles, which are referred to as non-Pythagorean, the lattice is aperiodic. Two examples are presented in Figure 2(b1,b2), corresponding to the superposition of two square lattices at Pythagorean angles Δ*α* = arctan (3/4) ≈ 36° (Δ*θ =* 18°) and Δ*α* = arctan (5/12) ≈ 22° (Δ*θ =* 11°), respectively. The resulting wavefields exhibit periodic moiré lattices and the period units are labeled with white squares. For non-Pythagorean angles, Δ*θ =* 14° and 9° in Figure 2(b3,b4), the generated wavefields exhibit an aperiodic structure (not disordered) and break the translational symmetry of the periodic lattices. However, they have a long-range order and preserve the four-fold rotational symmetry of lattices.

**Figure 3:**
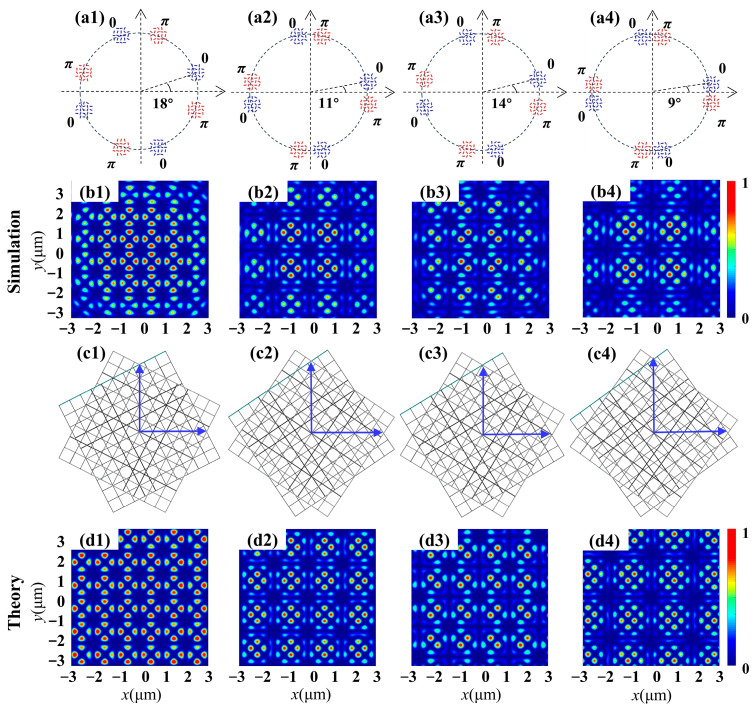
Moiré lattice wavefields generated by the superposition of two twisted square lattices with phase differences Δ*φ* = π. (**a1**–**a4**) Schematic diagrams of the metasurface sample depict two sets of 4 + 4 circular apertures with axes mutually rotated by the angle indicated in each figure, distinguished by blue and red labels. (**b1**–**b4**) Simulated intensity profiles of moiré lattices produced by the interference of two square patterns. (**c1**–**c4**) Schematic discrete representation of two rotated square sublattices. (**d1**–**d4**) Theoretical results of intensity distributions.

Following the superposition of two square lattices with identical phases, the next types of metasurfaces were designed to perform the superposition in opposite phases. The wavefields of the four metasurfaces were simulated, each of which contained two sets of four round apertures with the same twist angles as mentioned above. The initial slit orientation angle in each aperture was set as *φ_j0_* = 0 and *φ_j0′_* = π/2, allowing for the establishment of a superposition of two square lattices with a phase difference Δ*φ* = π. The schematic diagrams of these metasurfaces are shown in the first row of Figure 3. The simulated and theoretical intensity images for all moiré lattices are presented in the second and last rows, respectively. It is noteworthy that a central dark point emerges in the resulting intensity patterns due to destructive interference, which is distinctly different from the centrally focused spot produced by superposition with an identical phase, as illustrated in Figure 2. By adjusting the twist angle between the two sets of four round apertures, diverse period patterns can be formed surrounding the central dark point. In the case of the first metasurface with a twist angle Δ*θ =* 18° and under RCP incidence, the intensity distribution is illustrated in Figure 3(b1), where the coherence between two simple square lattice fields with inverse phases results in an intensity pattern characterized by periodic loops. Each loop comprises eight bright spots surrounding the central point. When the phase difference remained unchanged at Δ*φ =* π but the twist angle was set at Δ*θ =* 14°, 11° and 9°, respectively, three distinct types of moiré lattices were produced, as shown in Figure 3(b2–b4). From these results, it is demonstrated that the periodic characteristics and the rotational symmetry will not be changed if the initial phase of each aperture is modulated.

**Figure 4:**
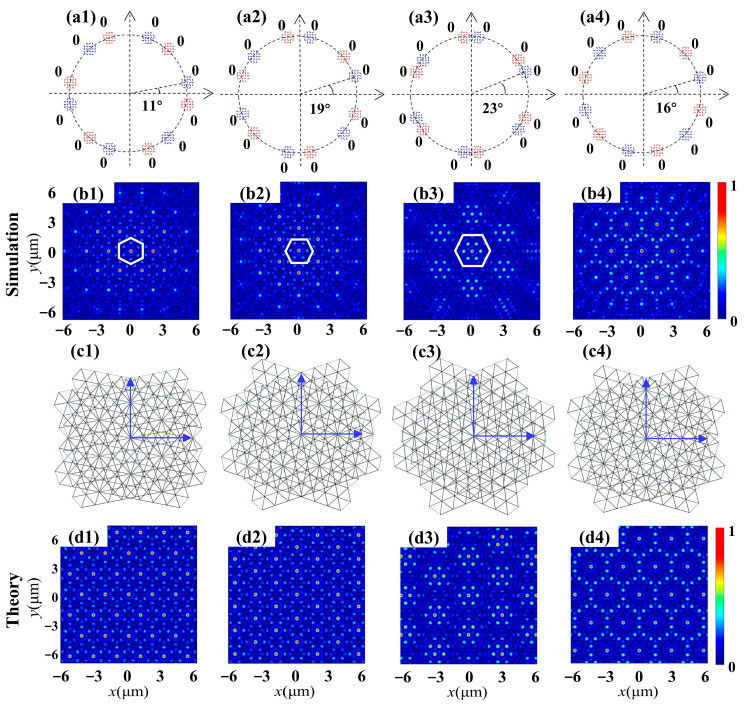
Moiré lattice wavefields generated by the superposition of two twisted hexagonal lattices with identical phases Δ*φ* = 0. (**a1**–**a4**) Schematic diagrams of the metasurface sample depict two sets of 6 + 6 round apertures with axes mutually rotated by the angle indicated in each figure, distinguished by blue and red labels. (**b1**–**b4**) Simulated intensity profiles of moiré lattices produced by the interference of two hexagonal patterns. (**b1**–**b3**) Periodic lattices with periodic units labeled with white hexagons. (**b4**) Aperiodic lattice. (**c1**–**c4**) Schematic discrete representation of two rotated hexagonal sublattices. (**d1**–**d4**) Theoretical results of intensity distributions.

Furthermore, the metasurfaces’ capability to support the superposition of two hexagonal lattices was investigated. For such lattices, the rotation angles producing period patterns are given by the relation tan (Δ*α*) = *b* √3/(2*a* + *b*), where the integers *a*, *b* and *c* solve the Diophantine equation *a*^2^ + *b*^2^ + *ab* = *c*^2^. The third type of metasurface was designed with the parameters *m = m*′ = 2 and *φ_j0_* = *φ_j0′_* = 0, grouped into two sets of six apertures, capable of constructing the superposition of two hexagonal lattices in phase difference Δ*φ* = 0. The incident light is an RCP vortex beam with TP *l* = 4. The schematic diagrams of four metasurfaces are shown in the first row in Figure 4. The corresponding simulated intensity patterns of the generated moiré lattices are illustrated in the second row, while the third row depicts their corresponding discrete moiré lattices. The simulated intensity profiles reveal distinct periodicity or exhibit a rotational symmetric feature. Furthermore, it should be noted that the intensity distribution is highly dependent on the Δ*θ* value. For Δ*θ* = 11°, which satisfies the Diophantine principle, the coherence of two simple hexagonal lattice fields with identical phases forms a periodic intensity pattern. Each unit exhibits a central focus spot and six alternating lobes in the outer circle. Interestingly, when comparing Figure 4(b1) with Figure 4(b2), with only slight azimuthal bias around approximately 30°, both moiré fields’ structures coincide. As Δ*θ* further increases to 23°, the hexagonal lattices emerge as prominent features with a period unit pattern, as depicted in Figure 4(b3). When the value of the twist angle fails to observe the Diophantine principle, in Figure 4(b4) with Δ*θ* = 16°, it exhibits an aperiodic structure (not disordered) and breaks the translational symmetry of the periodic lattices. The aforementioned alterations demonstrate that even slight adjustments in the twist angle can result in distinct transformations within moiré patterns. All of the intensity profiles have good consistency with the theoretical results, as shown in the last row of Figure 4, verifying the highly efficient generation of different moiré lattices with the designed metasurfaces.

**Figure 5:**
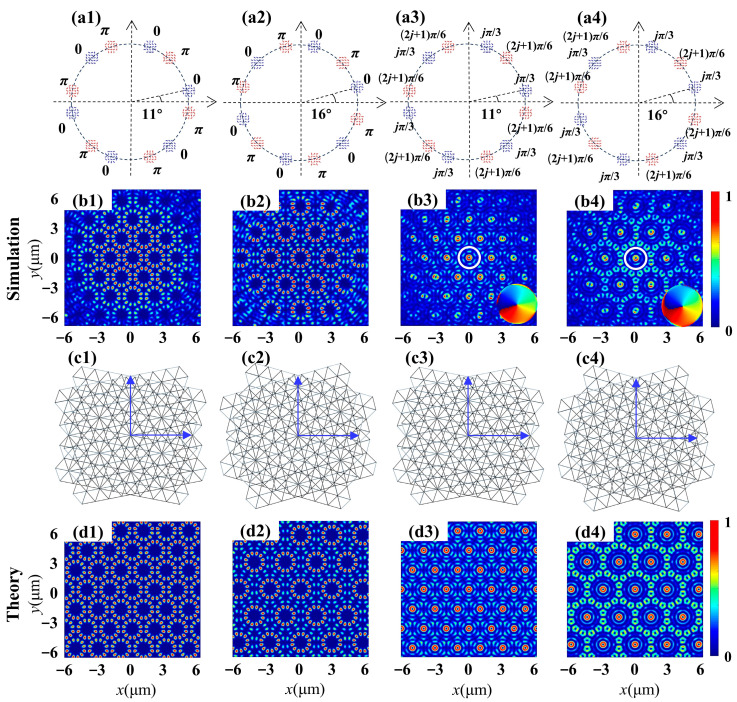
Moiré lattice wavefields generated by the superposition of two twisted hexagonal lattices with varying phase differences. (**a1**–**a4**) Schematic diagrams of the metasurface sample depict two sets of 6 + 6 round apertures with axes mutually rotated by the angle indicated in each figure, distinguished by blue and red labels. (**b1**–**b4**) Simulated intensity profiles of moiré lattices produced by the interference of two hexagonal patterns. The insets show the corresponding phase distribution. (**c1**–**c4**) Schematic discrete representation of two rotated hexagonal sublattices. (**d1**–**d4**) Theoretical results of intensity distributions.

The subsequent design involves a fourth type of metasurface, aimed at generating a superposition of two hexagonal lattices with varying phase differences. Four metasurfaces were designed and implemented for simulation and theoretical calculation. Figure 5 illustrates the intensity distribution corresponding to the generated moiré lattice wavefield for the illumination of an RCP vortex beam with TP *l* = 4. Initially, we designed two metasurfaces with twist angles of Δ*θ* = 11° and 16°, respectively, to demonstrate the superposition of two hexagonal lattices with reversed phases. The parameters are identical to those of the third type of metasurface, with the exception of the initial angles at *φ_0_* = 0 and *φ_0′_* = π/2 for each aperture. The moiré lattice field with period distribution, as shown in Figure 5(b1,b2), exhibits the expected six-fold symmetric feature. In comparison with the results achieved through the in-phase superposition of two hexagonal lattices in Figure 4(b1,b2), both fundamental period units and moiré lattice fields have undergone modification due to the introduction of a phase difference π. For instance, when Δ*θ* = 11°, Figure 5(b1) illustrates the intensity distribution of lattices where each unit is represented by a ring composed of twelve discrete spots. It is noteworthy that each unit of the generated moiré lattices is similar to those found in the central part of 12-fold symmetric quasiperiodic wavefields. Similarly, the superposition of two hexagonal lattices with π phase differences is also conducted using the second metasurface with Δ*θ* = 16°. The generated intensity pattern closely resembles that in Figure 4(b1), but the area covered by the period unit increases and forms two rings with twelve discrete spots. Furthermore, the proposed scheme can also be extended to manipulate the superposition with more intricate phase differences. For the third metasurface sample with Δ*θ* = 11°, *φ_0_* = *j*π/3 and *φ_0′_* = (2*j* + 1) π/6, a vortex with one topological charge (demonstrated by the phase pattern in the inset) is generated at the center of every unit cell, as shown in Figure 5(b3). Figure 5(b4) presents the intensity distribution of the generated vortex moiré lattice wavefield when Δ*θ* is increased to 16°, which contains an identical vortex with one topological charge in the center and twelve vortices in the outer circle. The intensity profiles of all the samples exhibit excellent consistency with the theoretical results, as demonstrated in the last row, confirming the highly efficient generation of diverse moiré lattices using the designed metasurfaces.

Although our work primarily focuses on the theoretical principles and design methods of metasurfaces, they have been validated through rigorous FDTD simulations and theoretical calculations. Here, the possible experimental measurements and demonstrations are suggested, which would not be too difficult to realize [42]. Regarding the experimental performance, a flat quartz with a thickness of 0.5 mm could be used as a substrate, on which a 200 nm thick gold film could be deposited by means of magnetron sputtering. The layouts of metasurface samples are exported as picture formats and then the pictures are imported to the program. Then, the sampling nanoslits may be etched in the gold film using a focused ion beam etcher (FIB). An optical configuration similar to that described in Reference [41] could be employed for the measurement of lattice fields produced by the fabricated samples, wherein a laser with a wavelength of 632.8 nm, in conjunction with a quarter-wave plate and a spiral phase plate, is utilized to generate circularly polarized vortex light. This light is employed to illuminate the sample from its substrate side. The intensity patterns of the moiré lattices are amplified using a microscope objective lens and subsequently captured by a scientific complementary metal-oxide semiconductor (s-CMOS) camera. The pinhole filter, comprising a quarter-wave plate and linear plate, is positioned in front of the s-CMOS camera to selectively filter out the converted CP component of the moiré lattices. A pinhole spatial filter was employed to generate a uniform spherical wave as a reference beam, which subsequently interfered with the beam. The s-CMOS camera captured both intensity and interference patterns, facilitating the reconstruction of phase distribution for the imaged lattices.

## 4. Discussion

For further discussion, we assume that the sum of the copolarization components of the nanoslits in the focal plane of the metasurface is approximately zero. This is achieved by introducing spin splitting and deflection by breaking the rotational symmetry of the metasurface. The cross-spin component, which carries a geometric phase related to the rotation angle of the nanoslit, is primarily considered for generating wavefields. By modulating the geometric rotation of each individual nanoslit, the geometric phase carried by cross-polarization components can be manipulated and modified in a flexible manner. The modulation of the rotation angle of the nanoslit facilitates the focus function in the metasurface, accompanied by diverse phase distribution within the circle area and distinct phase differences between two sets of round apertures. Manipulating the superposition of two square or hexagonal lattices with different twist angles and phase differences enables a wide range of diverse moiré patterns. Depending on the twist angle, the photonic moiré lattice exhibits different periodic or aperiodic structures. Our approach possesses several practical and technical advantages over other methods for producing period photonic lattices, such as computer holography [29], polygon excitation slits [40], moiré nanolithography [43] and optical bilayer photonic crystals [44]. Table 1 briefly compares the functionality and disadvantages of the proposed visualization techniques. In fact, the intensity profiles of moiré lattices can also be influenced by the relative amplitude of two sublattices. The primary focus of this work lies in demonstrating the functionality of phase modulation in metasurfaces through the equal weighting of the superposition. The capabilities of these metasurfaces can be enhanced by adjusting the radius and nanoslit distribution period of each round aperture, enabling amplitude modulation of two lattices and achieving the unequal weighting of the superposition.

## 5. Conclusions

In summary, a strategy was proposed for controlling the superposition of two sublattices with varying twist angles and phase differences in the plasmonic metasurface to generate a concentrated moiré lattice at the subwavelength scale. The proposed metasurfaces manipulate the geometric phase by controlling the orientation angle of each nanoslit, enabling the flexible generation and superposition of sublattices with different twist angles and phase differences. Four types of metasurfaces were developed to facilitate the superposition of two square or two hexagonal lattices. This innovative approach enables the creation of a diverse array of moiré lattices that exhibit period intensity patterns. A comprehensive analysis was conducted to investigate the generation of focused moiré lattices, and the numerical simulations were carried out using FDTD solutions. The numerically calculated results demonstrated remarkable agreement with the theoretical models proposed in this study. Our metasurface design provides a novel method for manipulating focused photonic moiré lattices flexibly and feasibly. The utilization of photonic moiré patterns provides an opportunity to explore the topological properties of light and to investigate phenomena that are relevant to other branches of physics, which may be challenging to directly explore, such as when manipulating ultracold atoms, creating gauge potentials and forming optical solitons, particularly condensed matter. The potential applications can also be expanded to atomic physics, specifically Bose–Einstein condensates.

## Figures and Tables

**Table 1:** The comparison between the generation methods of photonic period lattices.

Methods	Functionalities	Disadvantages
Computer holography	Can generate different moiré lattices.	(1) Require large-volume optical paths and complex algorithms. (2) Cannot manipulate the moiré lattices at subwavelength.
Polygon excitation slit	Can generate optical lattices (such as honeycomb, Kagome and hexagonal lattices) at subwavelength.	Cannot achieve the superposition of two-period sublattices and generate moiré lattices.
Moiré nanolithography	Can acquire the superposition of two hexagonal lattices with identical phases.	Cannot realize the superposition of other sublattices like square lattices and hexagonal vortex lattices.
Optical bilayer photonic crystal	Can obtain the superposition of two square and hexagonal lattices with identical phases.	Cannot modulate the phase difference between two superposed lattices.

## Data Availability

Data are contained within the article.

## References

[1] Cao Y., Fatemi V., Fang S., Watanabe K., Taniguchi T., Kaxiras E., Jarillo-Herrero P. (2018). Unconventional superconductivity in magic-angle graphene superlattices. Nature.

[2] Cao Y., Fatemi V., Demir A., Fang S., Tomarken S.L., Luo J.Y., Sanchez-Yamagishi J.D., Watanabe K., Taniguchi T., Kaxiras E. (2018). Correlated insulator behaviour at half-filling in magic-angle graphene superlattices. Nature.

[3] Lu X., Stepanov P., Yang W., Aamir M.A., Das I., Efetov D.K. (2019). Superconductors, orbital magnets and correlated states in magic-angle bilayer graphene. Nature.

[4] Oh M., Nuckolls K.P., Wong D., Lee R.L., Liu X., Watanabe K., Yazdani A. (2021). Evidence for unconventional superconductivity in twisted bilayer graphene. Nature.

[5] Tseng C.C., Ma X., Liu Z., Watanabe K., Taniguchi T., Chu J.H., Yankowitz M. (2022). Anomalous Hall effect at half filling in twisted bilayer graphene. Nat. Phys..

[6] Geisenhof F.R., Winterer F., Seiler A.M., Lenz J., Xu T., Zhang F., Weitz R.T. (2021). Quantum anomalous Hall octet driven by orbital magnetism in bilayer graphene. Nature.

[7] Lin J.X., Zhang Y.H., Morissette E., Wang Z., Liu S., Rhodes D., Li J.I.A. (2022). Spin-orbit–driven ferromagnetism at half moiré filling in magic-angle twisted bilayer graphene. Science.

[8] Hu G., Ou Q., Si G., Wu Y., Wu J., Dai Z., Krasnok A., Mazor Y., Zhang Q., Bao Q. (2020). Topological polaritons and photonic magic angles in twisted α-MoO_3_ bilayers. Nature.

[9] Chen M., Lin X., Dinh T.H., Zheng Z., Shen J., Ma Q., Chen H., Jarillo-Herrero P., Dai S. (2020). Configurable phonon polaritons in twisted α-MoO_3_. Nat. Mater..

[10] López M.R., Peñaranda F., Christensen J., San-Jose P. (2020). Flat bands in magic-angle vibrating plates. Phys. Rev. Lett..

[11] Park M.J., Kim Y., Cho G.Y., Lee S. (2019). Higher-order topological insulator in twisted bilayer graphene. Phys. Rev. Lett..

[12] Sunku S.S., Ni G., Jiang B.Y., Yoo H., Sternbach A., McLeod A.S., Basov D.N. (2018). Photonic crystals for nano-light in moiré graphene superlattices. Science.

[13] Du Z., Yang S., Li S., Lou J., Zhang S., Wang S., Li B., Gong Y., Song L., Zou X. (2020). Conversion of non-van der Waals solids to 2D transition-metal chalcogenides. Nature.

[14] Domröse T., Danz T., Schaible S.F., Rossnagel K., Yalunin S.V., Ropers C. (2023). Light-induced hexatic state in a layered quantum material. Nat. Mater..

[15] Wang P., Zheng Y., Chen X., Huang C., Kartashov Y.V., Torner L., Ye F. (2020). Localization and delocalization of light in photonic moiré lattices. Nature.

[16] Tan Z., Fan F., Guan S., Wang H., Zhao D., Ji Y., Chang S. (2023). Terahertz Spin-Conjugate Symmetry Breaking for Nonreciprocal Chirality and One-Way Transmission Based on Magneto-Optical Moiré Metasurface. Adv. Sci..

[17] Hong P., Liang Y., Chen Z., Zhang G. (2023). Robust moiré flatbands within a broad band-offset range. Adv. Photonics Nexus.

[18] Gómez-Urrea H.A., Ospina-Medina M.C., Correa-Abad J.D., Mora-Ramos M.E., Caro-Lopera F.J. (2020). Tunable band structure in 2D Bravais–Moiré photonic crystal lattices. Opt. Commun..

[19] Du L., Molas M.R., Huang Z., Zhang G., Wang F., Sun Z. (2023). Moiré photonics and optoelectronics. Science.

[20] Zeng J., Hu Y., Zhang X., Fu S., Yin H., Li Z., Chen Z. (2021). Localization-to-delocalization transition of light in frequency-tuned photonic moiré lattices. Opt. Express..

[21] Guan J., Hu J., Wang Y., Tan M.J., Schatz G.C., Odom T.W. (2023). Far-field coupling between moiré photonic lattices. Nat. Nanotechnol..

[22] Hu G., Krasnok A., Mazor Y., Qiu C.W., Alù A. (2020). Moiré hyperbolic metasurfaces. Nano Lett..

[23] Ivanov S.K., Konotop V.V., Kartashov Y.V., Torner L. (2023). Vortex solitons in moiré optical lattices. Opt. Lett..

[24] Gao Y., Wen Z., Zheng L., Zhao L. (2017). Complex periodic non-diffracting beams generated by superposition of two identical periodic wave fields. Opt. Commun..

[25] Lowell D., Hassan S., Sale O., Adewole M., Hurley N., Philipose U., Chen B., Lin Y. (2018). Holographic fabrication of graded photonic super-quasi-crystals with multiple-level gradients. Appl. Opt..

[26] Huang L., Zhang W., Zhang X. (2022). Moiré quasibound states in the continuum. Phys. Rev. Lett..

[27] Salakhova N.S., Fradkin I.M., Dyakov S.A., Gippius N.A. (2023). Twist-tunable moiré optical resonances. Phys. Rev. B.

[28] Nguyen D.X., Letartre X., Drouard E., Viktorovitch P., Nguyen H.C., Nguyen H.S. (2022). Magic configurations in moiré superlattice of bilayer photonic crystals: Almost-perfect flatbands and unconventional localization. Phys. Rev. Res..

[29] Shang C., Lu C., Tang S., Gao Y., Wen Z. (2021). Generation of gradient photonic moiré lattice fields. Opt. Express..

[30] Sale O., Hassan S., Hurley N., Alnasser K., Philipose U., Zhang H., Lin Y. (2020). Holographic fabrication of octagon graded photonic supercrystal and potential applications in topological photonics. Front. Optoelectron..

[31] Park J.S., Zhang S., She A., Chen W.T., Lin P., Yousef K.M., Capasso F. (2019). All-glass, large metalens at visible wavelength using deep-ultraviolet projection lithography. Nano Lett..

[32] Wang C., Sun Y., Yu Z., Liu X., Chen B., Zhang Y., Zheng Z. (2023). Dual-Functional Tunable Metasurface for Meta-Axicon with a Variable Depth of Focus and Continuous-Zoom Metalens. Nanomaterials.

[33] Ren H., Briere G., Fang X., Ni P., Sawant R., Héron S., Genevet P. (2019). Metasurface orbital angular momentum holography. Nat. Commun..

[34] Li Z., Zhang Y., Yuan J., Hong Y., Liu H., Guo J., Wei Z. (2022). Three-Channel Metasurfaces for Multi-Wavelength Holography and Nanoprinting. Nanomaterials.

[35] Liu W., Li Z., Li Z., Cheng H., Tang C., Li J., Chen S., Tian J. (2019). Energy-Tailorable Spin—Selective Multifunctional Metasurfaces with Full Fourier Components. Adv. Mater..

[36] Solntsev A.S., Agarwal G.S., Kivshar Y.S. (2021). Metasurfaces for quantum photonics. Nat. Photonics.

[37] Gao H., Li Y., Chen L., Jin J., Pu M., Li X., Gao P., Wang C., Luo X., Hong M. (2018). Quasi-Talbot effect of orbital angular momentum beams for generation of optical vortex arrays by multiplexing metasurface design. Nanoscale.

[38] Zhang R., Zhang Y., Ma L., Zeng X., Li X., Zhan Z., Cheng C. (2019). Nanoscale optical lattices of arbitrary orders manipulated by plasmonic metasurfaces combining geometrical and dynamic phases. Nanoscale.

[39] Tsesses S., Ostrovsky E., Cohen K., Gjonaj B., Lindner N.H., Bartal G. (2018). Optical skyrmion lattice in evanescent electromagnetic fields. Science.

[40] Tsesses S., Cohen K., Ostrovsky E., Gjonaj B., Bartal G. (2019). Spin–orbit interaction of light in plasmonic lattices. Nano Lett..

[41] Zhang R., Gu M., Sun R., Zeng X., Zhang Y., Zhang Y., Cheng C. (2022). Plasmonic metasurfaces manipulating the two spin components from spin–orbit interactions of light with lattice field generations. Nanophotonics.

[42] Zhang Y., Zhang R., Li X., Ma L., Liu C., He C., Cheng C. (2019). Radially polarized plasmonic vector vortex generated by a metasurface spiral in gold film. Opt. Express.

[43] Lubin S.M., Zhou W., Hryn A.J., Huntington M.D., Odom T.W. (2012). High-rotational symmetry lattices fabricated by moiré nanolithography. Nano Lett..

[44] Tang H., Lou B., Du F., Zhang M., Ni X., Xu W., Mazur E. (2023). Experimental probe of twist angle–dependent band structure of on-chip optical bilayer photonic crystal. Sci. Adv..

